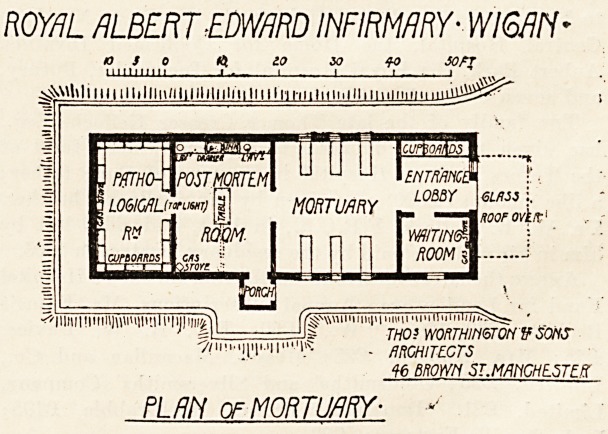# Royal Albert Edward Infirmary, Wigan, New Mortuary

**Published:** 1910-10-29

**Authors:** 


					ROYAL ALBERT EDWARD INFIRMARY,,
WIGAN, NEW MORTUARY.
This little building, which has just been erected fromr
the design of Messrs. Thomas Worthington and Sons, of
Manchester, contains a waiting-room, mortuary, post-
mortem room, and pathological-room. The entrance for
friends and for the undertaker is at the end of the build-
ing, and a glass roof forms a covered porch for shelter to
a hearse. The mortuary chamber itself is planned for six
bodies, and a chapel-like appearance is obtained by means
of a curved ceiling and lancet windows filled with stained
glass. It is to be regretted, however, that the authorities.
ROYAL ALBERT EDWARD INFIRMARY-W16AM <
tO 3 0 10 50 +0 50FJ
milium i .   i   . i i .. . ? ^
mi 1111H1 u/111U1111) I n 11II11 ii ri u < 11 n 11II111 m r ii m i
' otter* r
f m(H \P05TM0KTLM\
I LOmU^m) U MORTUARY
M J mm I n n n
^|llnil!llllll)WH||l!IHMI||l|ll4^ fJ^wnuiiiiuiM'iiiMi 'uuniiiiiiiTTiSS^. ?
1 ^I1 'is* THO? WOftTHIWoTOH ti1 SOUS'
. ARCHITECTS
i6 BHOWti ST.MANCHESTER
PLAN of MORTUARY- ?*'
did not go a step further and provide a separate room inr
which one body at a time could be reverently disposed for
friends to see. Such an arrangement would not have in-
creased the size of the building very much, and would
certainly be more in accordance with modern ideas of
decency and fitness. The post-mortem room communicates
directly with the mortuary, and has also a porch entrance
from the outside. The pathological-room would appear
to be intended more as a museum than as a laboratory, all
four sides being lined with cupboards and no working
bench being shown. This room is lighted from the top
only, a form of lighting which is practically useless for
microscopic work.

				

## Figures and Tables

**Figure f1:**